# Evaluating the accuracy of a new robotically assisted system in cadaveric total knee arthroplasty procedures

**DOI:** 10.1186/s13018-024-04788-8

**Published:** 2024-06-15

**Authors:** Jiafeng Yi, Zhisen Gao, Yijian Huang, Yubo Liu, Yiling Zhang, Wei Chai

**Affiliations:** 1 Senior Department of Orthopedics, The Fourth Medical Center of Chinese PLA General Hospital, Beijing, China; 2 School of Medicine, Nankai University, Tianjin, China; 3 National Clinical Research Center for Orthopedics and Sports Rehabilitation, Beijing, China; 4 Medical School of Chinese PLA, Beijing, China; 5 Longwood Valley Medical Technology Co. Ltd, Beijing, China

**Keywords:** Total knee arthroplasty, ROPA TKA system, Bone resection, Robotic surgery, Accuracy, Cadaveric study

## Abstract

**Background:**

Robot-assisted total knee arthroplasty (TKA) has been shown to facilitate high-precision bone resection, which is an important goal in TKA. The aim of this cadaveric study was to analyze the accuracy of the target angle and bone resection thickness of a recently introduced robotic TKA system.

**Methods:**

This study used 4 frozen cadaveric specimens (8 knees), 2 different implant designs, navigation, and a robotic system. The 4 surgeons who participated in this study were trained and familiar with the basic principles and operating procedures of this system. The angle of the bone cuts performed using the robotic system was compared with the target angles from the intraoperative plan. For each bone cut, the resection thickness was recorded and compared with the planned resection thickness.

**Results:**

The mean angular difference for all specimens was less than 1°, and the standard deviation was less than 2°. The mean difference between the planned and measured angles was close to 0 and not significantly different from 0 except for the difference in the frontal tibial component angle, which was 0.88°. The mean difference in the hip-knee-ankle axis angle was − 0.21°± 1.06°. The mean bone resection difference for all specimens was less than 1 mm, and the standard deviation was less than 0.5 mm.

**Conclusions:**

The results of the cadaveric experimental study showed that the new TKA system can realize highly accurate bone cuts and achieve planned angles and resection thicknesses. Despite the limitations of small sample sizes and large differences between cadaveric and clinical patients, the accuracy of cadaveric experiments provides strong support for subsequent clinical trials.

## Introduction

Osteoarthritis is one of the most common joint diseases among elderly people. With the growing and aging population in China, the demand for total knee arthroplasty (TKA), a classic procedure for the treatment of terminal osteoarthritis, is bound to increase annually [1]. The prevalence of osteoarthritis is 50% in people over 65 years old and up to 80% in people over 75 years old. Every year, 50,000 to 70,000 osteoarthritis patients undergo TKA to relieve pain and restore function. Despite great improvements in prosthetic design, surgical instruments, surgical techniques and postoperative rehabilitation in recent years, the rate of unsatisfactory outcomes after conventional TKA ranges from 10 to 20% due to errors in surgical planning, poor prosthetic positioning, and inaccurate force line recovery [2]. Pain, limited joint motion, joint instability and surgery-related complications are the most unsatisfactory symptoms for postoperative patients [3]. Inadequate intraoperative adjustments and gap balancing are the main causes of these limitations [4].

In recent years, with the continuous development of robotics and navigation technology, orthopedic surgery robots have become increasingly accepted by the public. Three different robots are used in robotic surgery: active, semiactive and passive robots [5]. The main active robot currently used for joint replacement is the ROBODOC (Think Surgical, USA), in which a robotic device independently performs the planned osteotomy, with the surgeon supervising the osteotomy handle to activate an emergency deactivation switch when needed. However, its high equipment cost and surgical complication rate during the learning phase of the procedure limit its application. Park et al. reported that the fully active robotic TKA system had a high short-term complication rate (6/32,18.8%) [6]. Semi-active robotic systems enable surgeons to maintain overall control over bone resection and implant positioning, providing real-time intraoperative feedback to limit deviations from the preoperative surgical plan. Rapid or violent movements could deactivate the robotic device to avoid bone and soft tissue injuries. The Mako robot system from the USA is a typical example of a semi-automatic robot. Passive system performs a portion of the surgery under the continuous and direct control of the surgeon. Orthopedic robots play an increasingly important role in preoperative planning, intraoperative positioning and prosthesis placement in TKA [7]. Recent clinical studies have shown that robot-assisted TKA can improve the accuracy of intraoperative osteotomy and postoperative prosthetic positioning and thus restore the postoperative force line [8, 9]. Compared with computer-assisted TKA, conventional TKA is poorly aligned, with 28–85% of cases achieving a deviation in mechanical leg alignment deviation within 3° varus/valgus, whereas computer-assisted alignment can achieve a rate of 76-100% [10]. However, their high cost, unique operating systems and additional radiological risks limit their large-scale application.

Currently, the domestic application of orthopaedic surgical robots in China is still at an early stage. The most widely used robotic system in the domestic market is the MAKO robotic system, which is based on a semiactive closed platform from Stryker Corporation in the USA. The FDA approved MAKO for TKA in 2008 and approved it for total hip arthroplasty (THA) in 2010 [11]. In TKA, studies have shown that MAKO offers greater accuracy and reproducibility in prosthetic position planning, osteotomy volume control, gap balancing, and lower limb force line restoration [12, 13]. However, the MAKO robot is very expensive ($800,000-$1200,000) [14]. Robot-assisted TKA has been shown to reduce the use of analgesic medications and stay in the hospital, and to have a lower rate of surgical complications compared to conventional TKA. However, the cost reductions in these areas are still not enough to overcome the supply costs of robotic surgery. Therefore the cost of robotic-assisted TKA remains high and time-consuming data transfers associated with the MAKO robot, limiting its use, particularly in developing areas [15].

In recent years, China’s domestic robots have also developed rapidly. The Bone Sheng Yuanhua Total Knee Replacement Assistive System (Yuanhua Robotics, Perception & AI Technologies Ltd., China), HURWA Knee Replacement Surgery Robotic System (Beijing HURWA Robotics Technology Co., Ltd.), and “SkyWalker” robotic system (MicroPort, China) have been verified through animal and cadaveric experiments, as well as multicentre randomized controlled trials completed at several large clinical centres. Therefore, these domestic robotic systems have promising prospects for application in TKA.

The ROPA TKA system (Longwood Valley MedTech) is a robotic system in early development with mature technology that can be used to assist in TKA in China. In preliminary work, the accuracy and stability of the ROPA TKA system have been verified with a large amount of data [16]. The system utilizes patient-specific lower limb computed tomography (CT) data, processed through an artificial intelligence-enhanced surgical planning program [17], which has been used in 34 provinces and cities and more than 600 tertiary hospitals across China, providing approximately 10,000 cases of artificial intelligence-planned results. Additionally, the interface of the ROPA TKA system is friendly and does not necessitate planning by engineers, thus reducing both the planning process and related cost. Finally, the surgical program planning process is efficient, and CT data can be directly imported to generate a planned program for the robot, which is convenient and efficient.

The purpose of this study was to determine the osteotomy accuracy of this newly designed robotic system for assisting in TKA. Therefore, the accuracy of the following measurements was analyzed relative to the target values in cadaveric experiments, as measured using a validated computer-assisted navigation system: the hip-knee-ankle axis (HKA) angle, the coronal frontal femoral component (FFC) angle, the frontal tibial component (FTC) angle, the femoral valgus angle (FVA) and the posterior tibial slope (PTS). This robotic system will accurately achieve preoperatively planned osteotomies, prosthesis placement and lower limb force lines. After each osteotomy, i.e., of the proximal tibial plateau, anterior and posterior condyles, and distal femur, the thickness of the cut was measured using a validated caliper. It was hypothesized that this robotic system would accurately achieve the bone resection, prosthesis placement and lower limb force lines consistent with the preoperative plan.

## Materials and methods

### Experimental specimens and main materials and instruments

The bilateral lower extremities of four adult cadaveric specimens (8 knees) with intact hip, knee, and ankle joints were used in this study. The cadavers were ethically sourced and free of knee-related diseases. The mean age at the time of death was 65.5 ± 3.35 years with a mean body mass index of 23.8 kg/m^2^. Two of the four cadavers were females. The knee implants used were sourced from Johnson & Johnson and ICON (two common prostheses used in TKA in China). The osteotomy plan was created according to the preoperative software of the ROPA TKA system, and the implant prosthesis was installed after the robot-assisted osteotomy procedure was completed. The four surgeons who participated in this study were knee arthroplasty specialists and who were familiar with the basic principles and operating procedures of the ROPA TKA system. This training consisted of a theoretical training on the functions of the ROPA TKA system, the practice of what was learnt during 3 total knee replacements performed on sawbones and 2 TKAs performed on cadavers before the beginning of the study. To standardize the protocol, the target HKA angle was 180°, with 90° for both the tibial and femoral coronal angles. The femoral valgus angle (FVA) was preoperatively set to 6° and the PTS was set to 3°. Then, the results were compared with those of the bone cuts performed using the robotic system. For each bone cut, the resection thickness was measured with a caliper 3 times by 2 different observers and compared with the planned resection value [18].

### Structure and working principle of the ROPA TKA robot

**Fig. 1:**
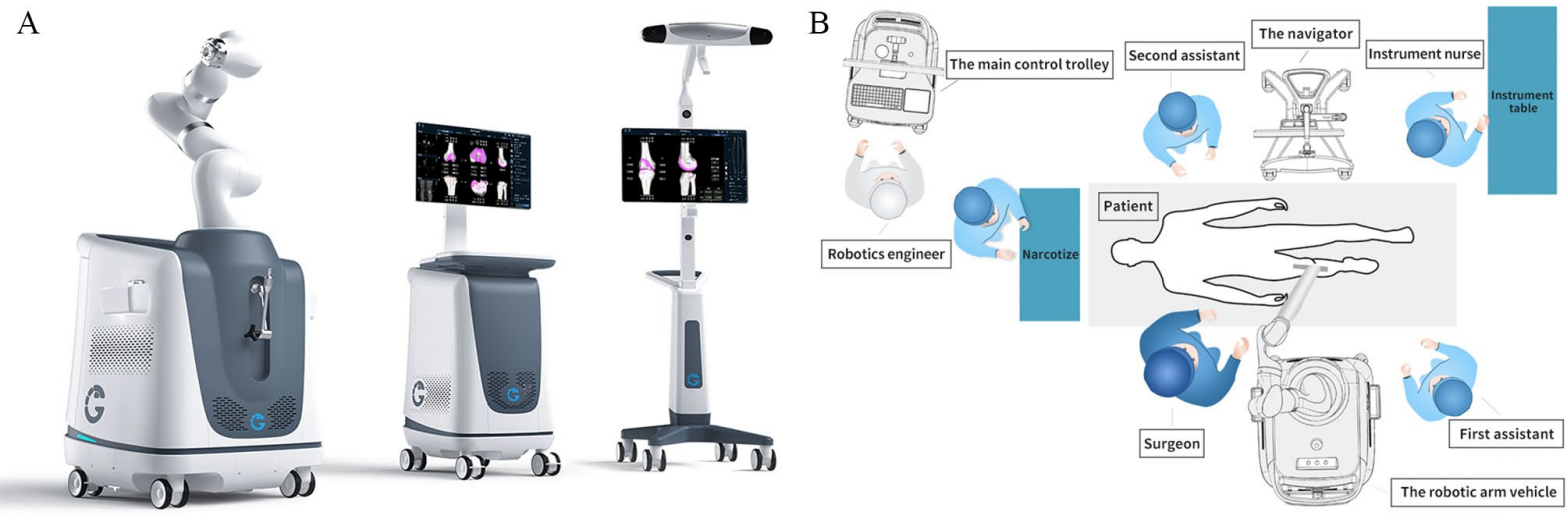
**(A)** The illustration of the ROPA TKA robot system. **(B)** The schematic diagram of the placement of the ROPA TKA robot system in the operating room

The ROPA TKA system consists of three parts: the navigator, the robotic arm vehicle and the main control trolley. Figure 1 shows the ROPA TKA robot system and the schematic diagram of its placement in the operating room. In the experimental process, the navigator recognizes and tracks the optical tracer and provides real-time feedback regarding the positions of the power tool and the surgical area to the main control trolley. The main control trolley is embedded in the software of the ROPA TKA system, which can complete the preoperative planning and merge the real-time data of the robotic arm trolley and navigator to execute intraoperative navigation algorithms. The end of the robotic arm trolley is connected to a power tool, and based on the navigational information, it recognizes the safe area for bone cutting. Based on the navigational information, the safe zone for osteotomy is identified, and the power and activity range of the power tool are restricted to prevent excessive osteotomy or accidental damage to the ligaments and other soft tissues around the knee joint, thus assisting the operator in completing precise and safe osteotomy operations.

### Robotic procedure

A 3D CT scan of each experimental cadaver was performed before the beginning of the experiment, and the obtained data were imported into the preoperative planning software of the ROPA TKA system in DICOM format. Then, CT segmentation and 3D reconstruction were performed to obtain a 3D model of the femur and tibia of the cadaver. Subsequently, osteotomy program planning was performed, including the angle and thickness of the osteotomies of the distal femur, anterior and posterior condyles and tibia, as well as the types of femoral and tibial prosthetic implants.

**Fig. 2:**
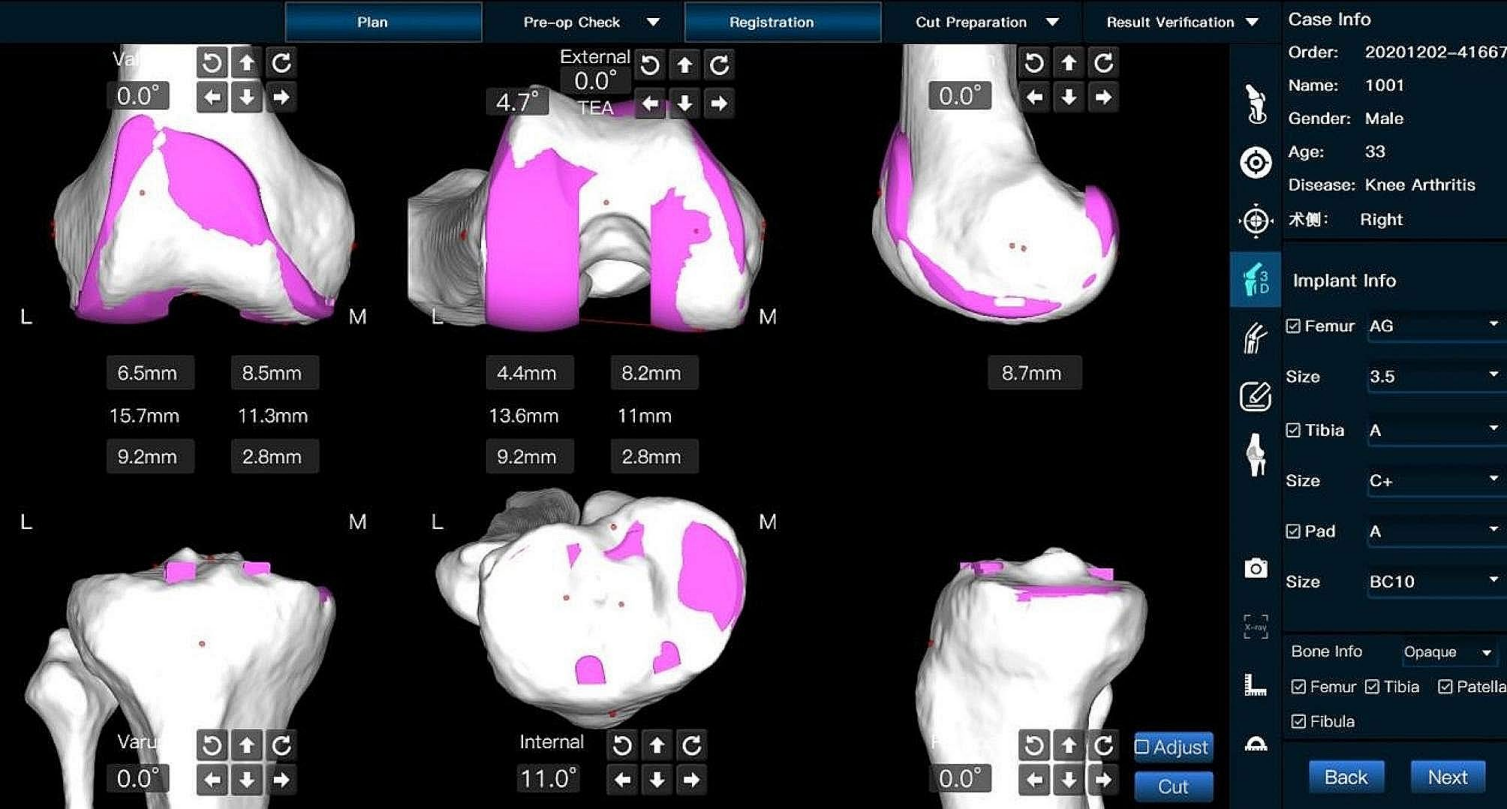
The operator performs preoperative planning using specialised software to determine the ideal resection thickness and angle to obtain a balanced and well-aligned TKA.

**Fig. 3:**
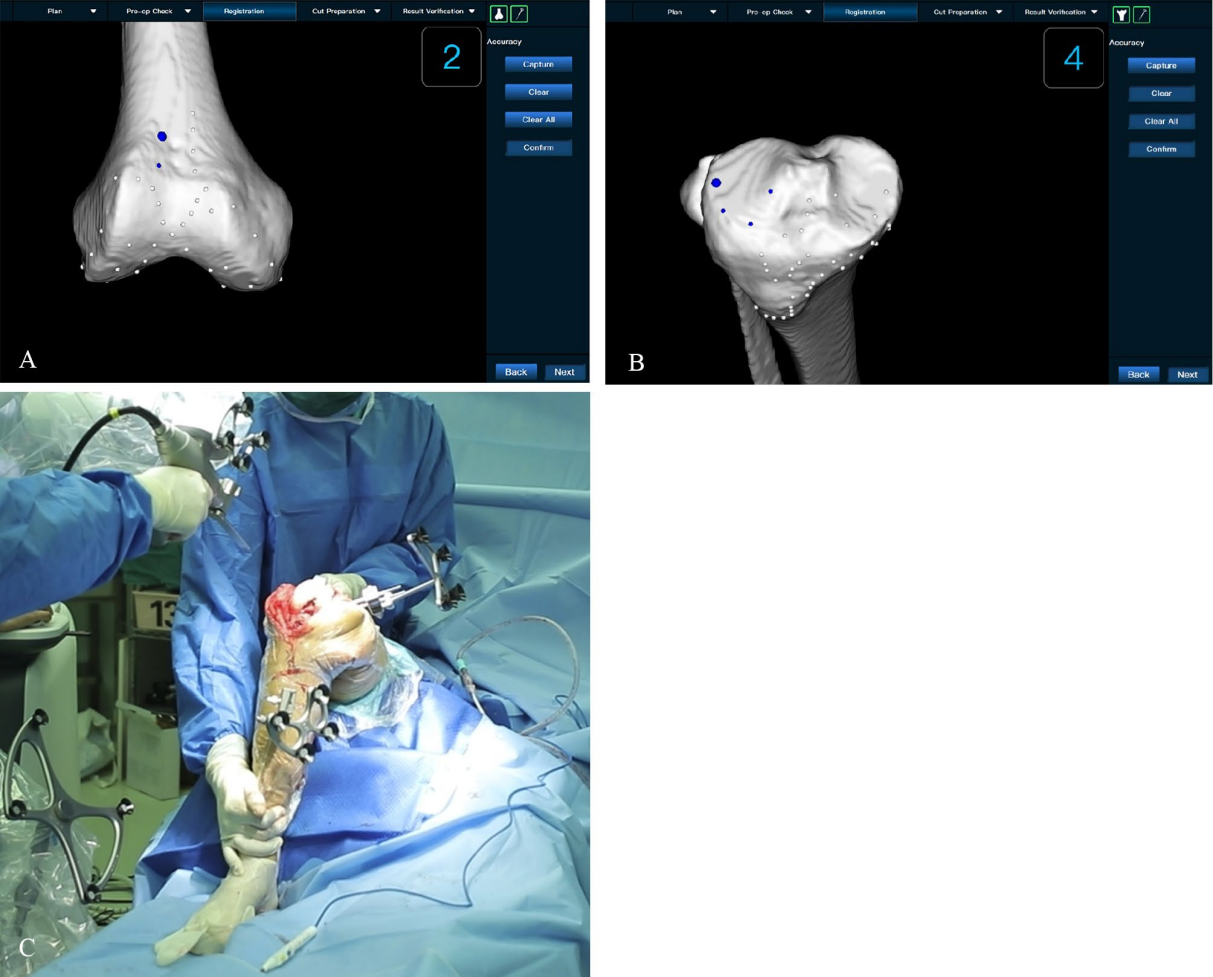
The schematic diagram of intraoperative calibration and osteotomy. **(A)** and **(B)**: The calibration of femoral and tibial on the ROPA TKA system. **(C)** The robotic arm of the ROPA TKA robotic system is fixed in the desired position determined by the surgical plan based on the operator’s intraoperative planning. Once the cutting jig is set and fixed in the correct position, the surgeon performs the cuts

**Fig. 4:**
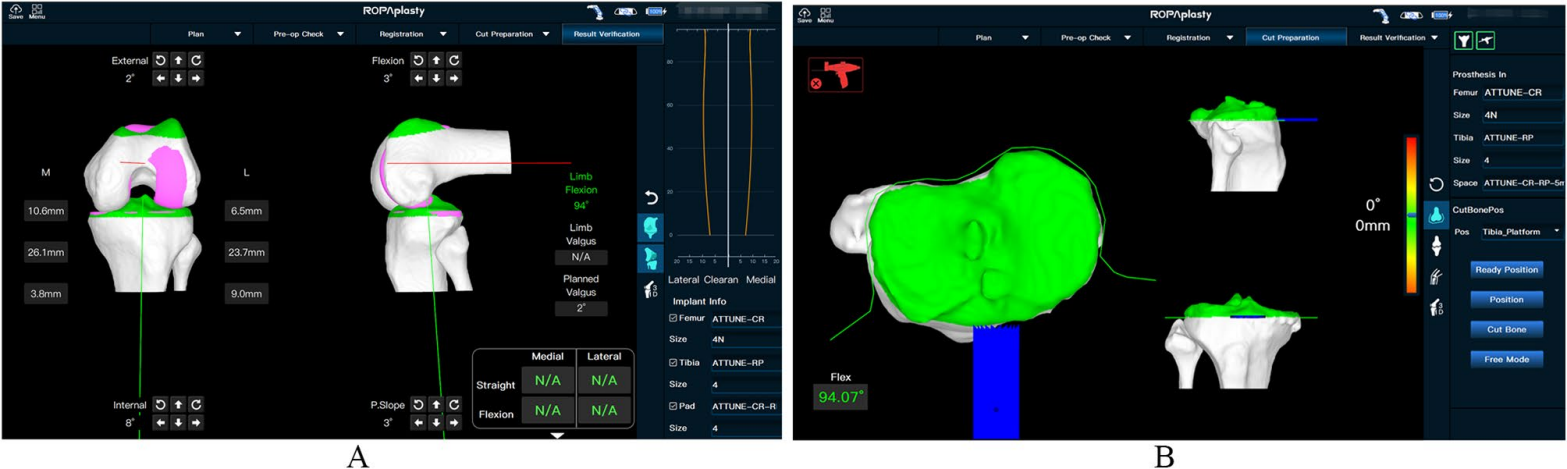
**(A)** The illustration of intraoperative adjustment of osteotomy parameters according to the soft tissue condition of the specimen. **(B)** The chematic diagram of intraoperative tibial osteotomy on the ROPA TKA system

Experimental procedure: The surgeon performed preoperative planning with AI three-dimensional preoperative planning software to automatically determine the ideal resection thickness and angle for a balanced and well-aligned TKA procedure. Figure 2 shows the preoperative planning for a cadaveric trial of the ROPA TKA system, which shows the planned femoral and tibial angles as well as the balanced gap. All cadaveric knee replacements were performed using the medial approach. The cadaveric specimen was fixed on the experimental table, and the skin and subcutaneous tissues were incised sequentially to fully reveal the distal femur, the anterior condyle and the tibial plateau. Two rigid body trackers were placed on each cadaveric knee, one on the femur and one on the tibia, to align the robot after the robot was calibrated. Figure 3 shows the installation of navigation on the femur and tibia of a cadaver in preparation for bone resection after the completion of calibration using the ROPA TKA system. The ROPA TKA system can display the relative positions of the femur and tibia as well as the osteotomy flexion and extension gaps in real time, at which point the surgeon can confirm the adjustment of the osteotomy parameters according to the soft tissue condition of the specimen. All planned angle and resection thickness values were recorded. The osteotomy robot was then used to perform the distal femoral cut first, followed by the tibial cut. Figure 4 shows the measurement of intraoperative gap and osteotomy thickness.

The ROPA assists doctors in performing intraoperative osteotomy operations through its osteotomy function. The position and angle information of the osteotomy surface are consistent with the preoperative plan, and the preoperative prosthesis planning osteotomy surface is accurately implemented through the positioning of the robotic arm. The software interface displays a total of 6 osteotomy surfaces for the femur and tibia (femoral anterior condyle, femoral anterior oblique, distal femur, femoral posterior oblique, femoral posterior condyle, and tibial plateau). Among them, the femoral anterior condyle, femoral posterior condyle, and tibial plateau provide safe boundary protection (to prevent damage to ligaments during the osteotomy process). During the osteotomy process, the software provides real-time feedback on the position and angle of the robotic arm’s movement, ensuring the visualization and accuracy of the intraoperative operation data. After completing femoral and tibial osteotomy. The prosthesis was fitted after the accuracy was confirmed, and the femoral and tibial tracers and fixation nails were then removed. Finally, the incision was closed.

### Measurement of the angle and resection thickness

The observers were trained in the method for measuring bone block thickness before the experiment and participated in intraoperative resection thickness measurements after stabilizing the measurements. The thickness of intraoperative resections was measured using a calibrated Vernier caliper (Mitutoyo, Japan). After each incision with the ROPA TKA system, the thickness of the resected bone was measured. Each cut was measured 3 times by 2 different independent observers. For each cadaver, the thickness of the cuts of the distal femur, anterior and posterior femoral condyles and proximal tibia was measured.

**Fig. 5:**
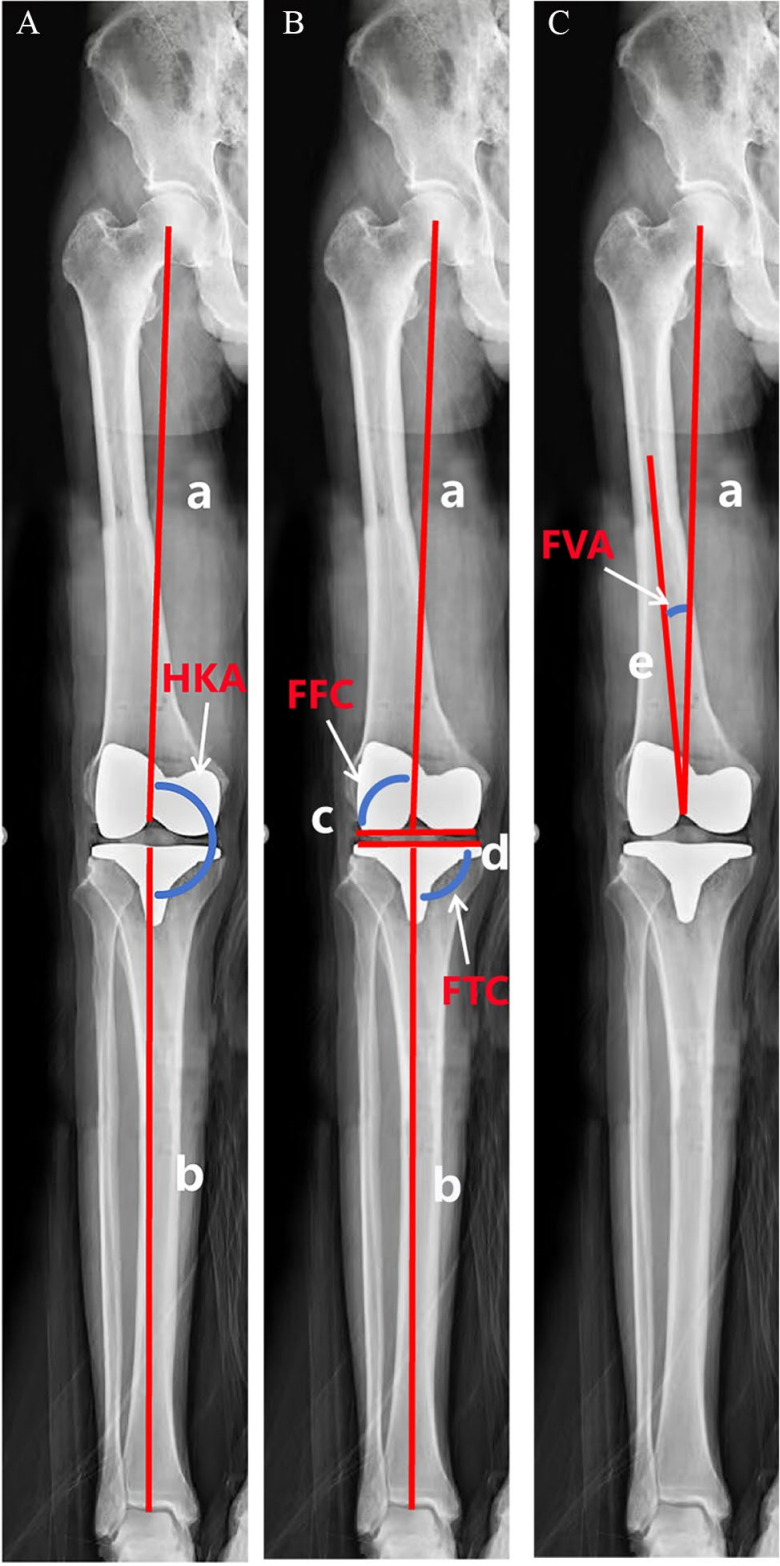
Measurement of HKA, FFC, FTC, FVA. **(A)** Line a was the femoral mechanical axis, and line b was the tibial mechanical axis; the medial angle formed between them was recorded as the HKA. **(B)** Line c was the line across the bottom of the femoral condyles, line d was the line across the bottom of the tibial plateau on the anteroposterior radiograph; the lateral angle between line a and line c was recorded as FFC, and the medial angle between line b and line d was recorded as FTC. **(C)** Line e was the anatomical axis of the femur, the acute angle between line a and line e was recorded as FVC.

To verify the accuracy of the prosthetic position, each specimen was examined by X-ray. The cadaveric specimen was placed in the lying position, with both lower limbs straightened, internally rotated by 15°, and the patella facing anteriorly. The joint position of the cadaveric specimen was fixed with sponge pads. Orthopantomographs of the hip, knee, and ankle joints were taken, and the DICOM files of the three radiographs were exported and merged to form a full-length radiograph of the lower limbs. Then, the image was imported into Image-Pro software, which was used to measure the postoperative HKA, FFC, FTC, and PTS. HKA is formed by lines connecting the centers of the femoral head, the knee and the talus. The FFC is the lateral angle between the femur mechanical axis and the line across the bottom of the femoral condyles. The FTC is the medial angle between the tibial mechanical axis and the line across the bottom of the tibial plateau. The PTS is the angle between the articular surface of the tibial plateau and the horizontal line on lateral X-ray of the lower limb. The detailed measurement schematic is shown in Fig. 5. To ensure measurement accuracy and reduce measurement error, two nonparticipating orthopedic surgeons with rich measurement experience obtained the measurements, and if the difference between two measurements was too -large (≥ 0.5°), a third nonparticipating orthopedic surgeon obtained the measurement, and the final result was taken as the mean of the two similar measurements.

### Statistical analysis

The data were analyzed using SPSS version 26 (IBM, Armonk, NY, USA). After the normality of the data was checked, descriptive statistics (mean, standard deviation and extreme deviation) were calculated. Continuous data are expressed as the mean and standard deviation. The proportion of differences within ± 1° and ± 2° was calculated for alignment values. Similarly, the proportions of differences within ± 1 mm and ± 2 mm were calculated for resection thicknesses. Hypotheses were proposed, and the difference between the ROPA robotic system navigation group and preoperative planning group was calculated to test whether the difference was normally distributed. When there were missing data or obvious outliers that had a large impact on the results, the obvious outliers in the data were removed, and replaced with the mean or median values. Paired t tests were selected for data with a normal distribution, and the signed rank sum test was selected for data with a skewed distribution. Conclusions were drawn based on the test P value. Confidence intervals of 95% were set a priori for both the t test and the 99% prediction interval. *P* < 0.05 was considered to indicate statistical significance.

## Results

**Fig. 6:**
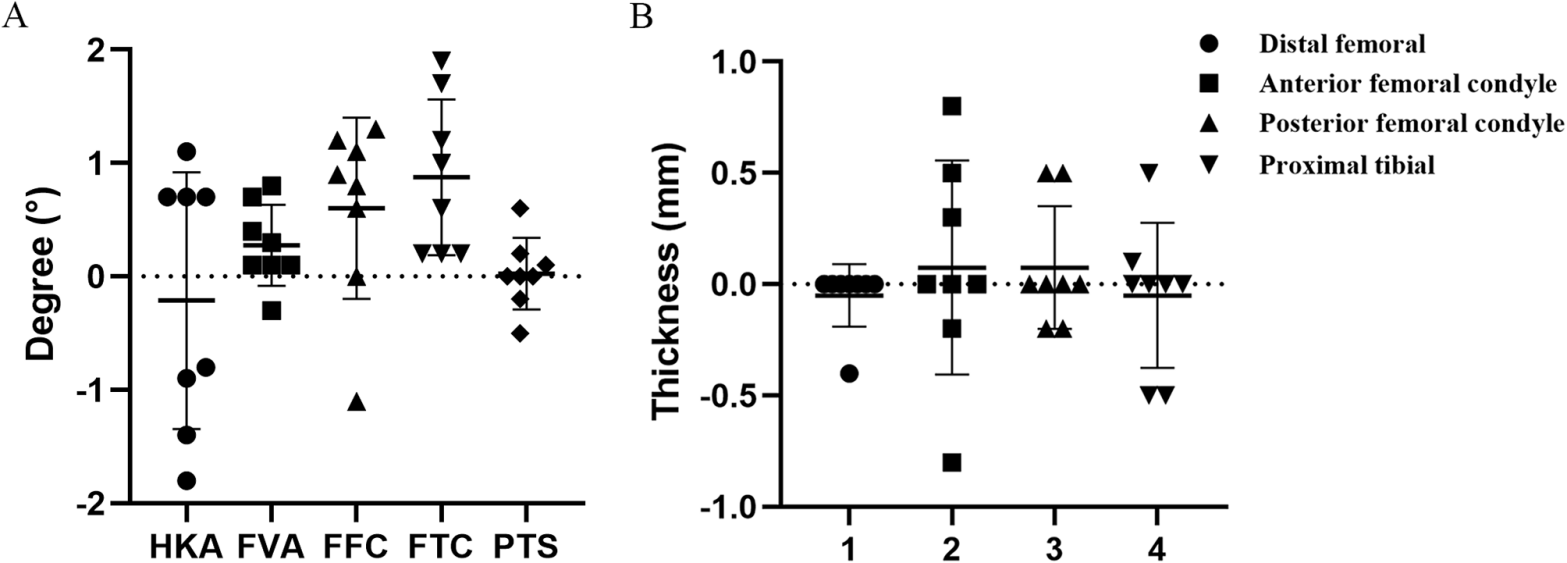
Graphical representation of angle **(A)** and thickness **(B)** differences

For all 8 specimens, the differences between the target and measured angles were found to follow a normal distribution, as shown in Table 1. Currently, the accuracy requirements of TKA robots, such as MAKO and ROSA, are generally within 1 mm and 1° [19, 20]. The osteotomy accuracy of the ROPA TKA system was evaluated by examining the angles within 1° and 2° in the cadaver experiments, as well as the resection thicknesses within 1 mm and 2 mm. In all cases, the mean difference was less than 1° (the maximum value was 0.88°), and the standard deviation was less than 2° (the maximum value was 1.06°). The mean difference between the planned and measured angles was close to 0 for all specimens and not significantly different from 0 except for the difference in the FTC angle, which was 0.88°. The P value of FTC was less than 0.05 (*P* = 0.009), which means that the difference was significant. Despite the significance of the P value for FTC, the mean value of the difference was 0.88°, and the difference in FTC was only clinically significant when it was greater than ± 3°. Similarly, all resection measurements are displayed in Table 2. For all specimens, the mean difference was less than 1 mm, and the standard deviation was less than 0.5 mm. The small standard deviation and mean values indicate that the ROPA TKA system could accurately realize the preoperative planning and the good stability of this system. For all measurements, the mean difference between the planned and measured resection thicknesses was not significantly different from 0. No outliers were observed any of the data. Although the standard deviation of the HKA is relatively large (SD = 1.06), its maximum difference is only −1.8°, which is within the normal range of the data. Figure 6 shows the specific distribution of the angle and thickness differences.

**Table 1:** Difference between planned angles and measured angles

Angles	Mean ± SD	*P* Value	Range (°)	% Within 2°	% Within 1°	99% PI
HKA	-0.21 ± 1.06	0.612	-1.8,1.1	100	62.5	-1.61, 1.19
FVA	0.28 ± 0.33	0.066	-0.3,0.8	100	100	-0.17, 0.72
FFC	0.6 ± 0.75	0.072	-1.1,1.3	100	50	-0.39, 1.59
FTC	0.88 ± 0.64	0.009	0.2,1.9	100	62.5	0.03,1.72
PTS	0.03 ± 0.29	0.829	-0.5,0.6	100	100	-0.36, 0.41

SD, standard deviation; Range, minimum to maximum; HKA, hip-knee-ankle angle; FFC, frontal femoral component; FTC frontal tibial component; FVA, femoral valgus angle; PI, predictive intervals

**Table 2:** Difference between planned bone resections and bone resections measured with the caliper

Parameters	Mean ± SD	*P* Value	Range (mm)	% Within 2 mm	% Within 1 mm	99% PI
Distal femoral	-0.05 ± 0.13	0.35	-0.4,0	100.00	100.00	-0.23, 0.13
Anterior femoral condyle	0.08 ± 0.45	0.67	-0.8,0.8	100.00	100.00	-0.52, 0.67
Posterior femoral condyle	0.08 ± 0.26	0.47	-0.2,0.5	100.00	100.00	-0.27, 0.42
Proximal tibial	-0.05 ± 0.3	0.68	-0.5,0.5	100.00	100.00	-0.45, 0.35

## Discussion

The hypothesis for this study was that this newly designed robotic system (ROPA TKA system) would achieve high accuracy, with an average error within 1° for angular values and within 1 mm for resection thickness values. The results showed that the cuts and angles measured with the ROPA TKA system were very accurate relative to the planned values, fully supporting this hypothesis. All angle errors were within 1° of each orther, with no significnt differences except for the FTC angle, which was 0.88°, and the P value was 0.009. This may be due to cadaveric osteoporosis, and the prosthesis may cause a change in the angle of the platform during implantation  [21]. This difference of FTC in this study remained within 1°, and according to the literature, a change within 3° does not affect the survival of the prosthesis [22, 23]. The mean difference in the HKA angle was calculated to be -0.21° ± 1.06°.

To verify the reliability and accuracy of the ROPA TKA system, this study involved a knee osteotomy experiment based on cadaveric lower limb specimens. The results showed that the ROPA TKA system was able to perform the entire cadaveric TKA procedure without any problems and the system could assist the operator in performing osteotomies according to the preoperative plan by helping control the osteotomy thickness and angle. In this experiment, the measured HKA angle and PTS values were within ± 2° of the preoperative values. Additionally, the errors between the measured and preoperatively planned osteotomy thicknesses were within 1 mm, which is also in line with the cadavetic study presented by Parratte et al. Parratte et al. evaluated the accuracy of a novel robotically assisted system for bone resection in total knee arthroplasty, utilizing Zimmer Biomet equipment. Results showed minimal mean differences and standard deviations below 1° for angle measurements, and below 0.7 mm for resection thickness. Despite industry funding and limited implant selection, rigorous methodology including intraoperative planning and computer-assisted measurements validated the system’s precision. These findings support the efficacy of robotic surgery in achieving precise outcomes comparable to conventional techniques. Further research is warranted to assess long-term clinical benefits and cost-effectiveness [20]. Therefore, the results of this study effectively verify the accuracy and safety of the ROPA TKA system for future clinical application.

Semi-active robotic systems allow surgeons to maintain overall control of bone resection and implant positioning while providing real-time intraoperative feedback to prevent deviation from the preoperative surgical plan, represented by MAKO (Stryker, USA) and ROSA Knee System(Zimmer Biomet, USA). Studies have shown that MAKO has greater accuracy and repeatability in planning the position of prostheses, controlling osteotomy, maintaining gap balance, and restoring lower limb force lines, as lower limb target force lines are still controversial [13, 24, 25]. The ROSA Knee System (Zimmer Biomet, USA) offers two options: imageless and image-based. The latter involves acquiring 2D X-rays and transforming them into a 3D model of the patient’s knee. Rossi et al. assessed the accuracy of the ROSA® Knee System by Zimmer Biomet, 75 TKA procedures were performed. Comparing planned, validated, and measured angles and cuts, statistically significant differences were observed only in femoral flexion, tibial coronal axis, medial, and lateral cuts, all remaining below 1 mm or under 1 degree with SD < 1. No differences were found between planned and measured cuts. The average difference in planned hip-knee-ankle (HKA) alignment was 1.2 ± 1.1, Correctly restored the lower limb force line [26]. These findings affirm the potential of robotic system to achieve accurate bone resections and align with planned angles in TKA.

However, in the realm of digital imaging technology, the application scope of three-dimensional preoperative planning is expanding. However, low-efficiency preparation remains a challenge. The segmentation of CT images by the MAKO robot is manually performed layer by layer, a complex process requiring a dedicated preoperative planning team and equipment. According to statistics, completing one manual preoperative planning session requires approximately 2 h of work from an experienced surgeon and a dedicated data processing engineer, adding significant manpower, resources, and time costs and greatly impeding the promotion of three-dimensional planning [27].

The introduction of artificial intelligence (AI) technology is key to addressing the issues of low efficiency and high costs associated with manual preoperative planning [28, 29]. Currently, there are reports abroad of the application of AI technology to knee joint CT data segmentation, but its accuracy and efficiency still fall short of clinical practicality [30]. Research teams at Siemens in the United States found the automatic segmentation of bony knee joint CT structures to be feasible, but there is still a gap in segmentation accuracy for clinical application. In China, although some joint robot development teams have developed three-dimensional preoperative planning, none have applied AI technology [31, 32]. The robot used in this study was the first in China to introduce AI technology into the field of three-dimensional preoperative planning and independently developed the first AI three-dimensional preoperative planning software product applicable for clinical use [16]. The ROPA TKA system used in this study was designed and optimized according to the needs of Chinese doctors. Therefore, it offers more user-friendly interactions and is more suitable for Chinese doctors’ operations and habits. First, the ROPA supports the modelling and adaptation of multiple systems and modeling of domestic and foreign prostheses, which is in line with the national conditions of China’s centralized procurement of prostheses. Second, the preoperative osteotomy simulation can be conducted after the confirmation of the planning scheme, which assists the operator in grasping the surgical scheme in a clearer and more three-dimensional way. At the same time, it is very convenient for surgical planning. The surgeon can import the patient’s CT data directly into the robot to plan and generate a surgical scheme, which is convenient and efficient.

This study also has several limitations. First, while the purpose of the study was to assess the accuracy of osteotomy, only the lower limb force line and femoral and tibial prosthetic angles were studied, which did not allow the relevant functions of the knee joint to be assessed. Thus, clinical studies need to be performed in osteoarthritis patients to further demonstrate the clinical applicability of the ROPA TKA robot system. Second, full-length radiographs of the lower extremities in the standing position could not be obtained in this study due to the limitations of the study subjects; therefore, three-joint orthopantomographs obtained in the supine position were spliced instead, which affects the accuracy and reliability of the experimental results to a certain extent. A third limitation is that we used cadaveric specimens, which typically exhibit less osteoarthritis and deformity than clinical cases. Despite the large gap between clinical cases and cadaveric specimens, the abilities of cadaveric specimens and clinical cases to evaluate surface prosthetic implantation accuracy are very similar. The next step of clinical research applied to osteoarthritis patients is to overcome these limitations.

The fourth limitation is the lack of measurements related to the rotational alignment of the implant in this study. The literature [33] has reported that the limited accuracy of rotation measurements in navigation systems and the error between interindividual and intraindividual variations during the palpation of the epicondyles can reach up to 6°. Rotation alignment is closely related to joint function after TKA [34], and alignment errors can damage the stability of the joint, alter tibiofemoral kinematics, and lead to patellar maltracking and pain. Implant cementation and three-dimensional postoperative CT scanning were not performed in this study to verify the rotation of the prosthesis because this would introduce a potential bias in the quality of fixation of uncemented and cemented implants, which is inconsistent with the ultimate goal of validating the accuracy of the robotic system for osteotomy. TKA in osteoarthritis patients may be helpful for addressing these limitations.

## Conclusion

Despite the inherent limitations of cadaveric experiments and the limited sample size in this study, the results of the angles and resection thicknesses show that the ROPA TKA system can assist the operator in planning accurate osteotomies and realizing the planned prosthetic placement position and angulation, which lays the foundation for the transition toward clinical application. Further in vivo studies are needed before clinical application, including cadaveric studies with expanded sample sizes to further validate the osteotomy accuracy of the ROPA system and in vivo studies in large animals to validate the effect of the ROPA TKA system on knee function. Moreover, it is necessary to investigate whether the accuracy observed in this cadaveric study can be replicated in clinical studies and to investigate other potential advantages of the system, such as time savings, optimization of implant positioning and improvements in patient-reported outcomes. Furthermore, the introduction of AI intelligent planning is expected to enable optimal individualized preoperative planning, which greatly improves the efficiency of robotic TKA surgery and is expected to be useful in a wider range of orthopedic procedures.

## Data Availability

The datasets generated during and/or analysed during the current study are available from the corresponding author on reasonable request.
